# Particulate Matter (PM) Research Centers (1999–2005) and the Role of Interdisciplinary Center-Based Research

**DOI:** 10.1289/ehp.11543

**Published:** 2008-09-15

**Authors:** Elinor W. Fanning, John R. Froines, Mark J. Utell, Morton Lippmann, Gunter Oberdörster, Mark Frampton, John Godleski, Tim V. Larson

**Affiliations:** 1 Center for Environmental and Occupational Health, School of Public Health, University of California at Los Angeles, Los Angeles, California, USA; 2 University of Rochester Medical Center, Rochester, New York, USA; 3 New York University School of Medicine, New York, New York, USA; 4 Department of Environmental Health, Harvard University School of Public Health, Boston, Massachusetts, USA; 5 Department of Civil and Environmental Engineering, University of Washington, Seattle, Washington, USA

**Keywords:** acute effects, biological mechanisms, chronic effects, criteria pollutants, dosimetry, exposure assessment, morbidity, mortality, particulate matter

## Abstract

**Objective:**

The U.S. Environmental Protection Agency funded five academic centers in 1999 to address the uncertainties in exposure, toxicity, and health effects of airborne particulate matter (PM) identified in the “Research Priorities for Airborne Particulate Matter” of the National Research Council (NRC). The centers were structured to promote interdisciplinary approaches to address research priorities of the NRC. In this report, we present selected accomplishments from the first 6 years of the PM Centers, with a focus on the advantages afforded by the interdisciplinary, center-based research approach. The review highlights advances in the area of ultrafine particles and traffic-related health effects as well as cardiovascular and respiratory effects, mechanisms, susceptibility, and PM exposure and characterization issues.

**Data sources and synthesis:**

The collective publications of the centers served as the data source. To provide a concise synthesis of overall findings, authors representing each of the five centers identified a limited number of topic areas that serve to illustrate the key accomplishments of the PM Centers program, and a consensus statement was developed.

**Conclusions:**

The PM Centers program has effectively applied interdisciplinary research approaches to advance PM science.

The U.S. Environmental Protection Agency (EPA) funded five academic centers in 1999 to address the uncertainties in exposure, toxicity and health effects of airborne particulate matter (PM) identified in the “Research Priorities for Airborne Particulate Matter” of the National Research Council (NRC 1998). Centers were established at Harvard University (Boston, MA), New York University (New York, NY), University of Rochester (Rochester, NY), University of Washington (Seattle, WA), University of California (Irvine, CA), University of California (Los Angeles, CA), and University of Southern California (Los Angeles, CA). All centers were structured to promote interdisciplinary approaches to address the research priorities of the NRC. A midterm report of PM Center findings was published previously (Lippmann et al. 2003). This report highlights selected accomplishments from the first 6 years of the PM Centers, with a focus on the advantages of interdisciplinary, center-based research. A more detailed summary of research findings and bibliography may be found in supplemental material available from the U.S. EPA PM Centers website (U.S. EPA 2008).

## PM Exposure Research Highlights

### Characterization of ambient PM

The PM Centers worked to characterize ambient PM and the substantial variation of concentration and composition with source, region, seasonal and diurnal patterns, and size fraction. Examples of these findings follow. In the eastern United States, PM_2.5_ (PM with aerodynamic diameter < 2.5 μm) composition varies seasonally, with relatively more sulfate from long-range transport in the winter, and nitrate in the summer. Substantial spatial variability in PM components and copollutants was observed (Maciejczyk and Chen 2005). In the Pacific Northwest, organic carbon (OC) derived from wood burning is a major contributor to fine particle mass (Larson et al. 2006). PM_10_ (PM < 10 μm in aerodynamic diameter) collected in Southern California derives largely from road dust and soil and contains significant quantities of metals, whereas PM_2.5_ from the same locations contains primarily nitrates, OC, and elemental carbon (EC). Ultrafine PM (UFP; PM < 0.1 μm in aerodynamic diameter) is especially high in OC (Sardar et al. 2005). Semivolatile components of PM have received increased attention in recent investigations, especially with regard to combustion-derived UFP in which a significant fraction of emissions by mass can consist of semivolatile material that has condensed onto a nonvolatile, primarily carbon core (Kuhn et al. 2005a; Robinson et al. 2007). Atmospheric processes generate UFP in regions of the Los Angeles, California, air basin that receive advected pollutant air masses (Fine et al. 2004; Singh et al. 2006). The role of atmospheric chemistry in formation of UFP is important: photo-oxidation of diesel emissions rapidly generates organic PM (Ntziachristos et al. 2007).

### Source apportionment

Research on sources emphasized mobile sources/traffic during the first 6 years of the PM Centers (see below). A workshop was held by the PM Centers to compare different methods for source apportionment of PM. The outcomes of different analytical methods found good agreement across different investigators and methods in apportioning sources of PM_2.5_ mass in two U.S. cities: Phoenix, Arizona, and Washington, D.C. (Hopke et al. 2006; Thurston et al. 2005). Center research also included identification of tracer compounds for use in identifying sources of ambient particles (Fine et al. 2004).

### Personal exposure

A significant body of data on personal exposure resulted from field studies of the PM Centers, including longitudinal studies conducted in different airsheds, populations, and housing. Extensive intra- personal and interpersonal variability in the ratio of personal to ambient exposure measures was observed in some studies (Liu et al. 2003), but taken collectively the data establish that ambient air concentrations at central site monitors can yield valid estimates of average personal exposure for population-based epidemiologic studies (Sarnat et al. 2000, 2002). The location of central site monitors, extent of PM penetration into indoor environments, personal activities, and the influence of indoor PM sources can affect personal/ambient exposure ratios (Larson et al. 2004; Sarnat et al. 2006). The effects of these factors differ with PM size and composition; for example, freeway-derived UFP in the 70- to 100-nm range penetrated indoors to a greater extent than 10- to 20-nm PM (Zhu et al. 2005). The relationship of ambient criteria pollutant concentrations to ambient and personal PM_2.5_ was explored. Ambient criteria pollutant levels were better predictors of personal PM_2.5_ than they were of personal exposure to the gaseous species themselves, suggesting that the criteria pollutants may be useful as surrogates of PM_2.5_ exposure, but are unlikely to act as confounders in epidemiologic studies (Sarnat et al. 2005). In a study of ambient UFP, hourly and 24-hr number concentrations were not significantly associated with concentrations of gaseous copollutants (Sardar et al. 2004).

## PM Health Effects and Mechanisms of Injury Highlights

During the effort of the U.S. EPA to establish a national ambient air quality standard for fine particles, considerable questions about the biological plausibility of epidemiologic findings on hospitalization and mortality from cardiopulmonary effects arose. As a result the NRC committee recommended research into the mechanisms of injury that underlie PM health effects, especially daily mortality. Developments in defining toxicologic mechanisms and intermediate clinical conditions that may explain the observed cardiovascular mortality are one of the highest impact areas of the scientific contributions of the PM Centers, in particular by addressing PM size-specific research, for example, ultrafine, fine, and coarse PM.

### PM effects on the cardiovascular system

**Figure 1:**
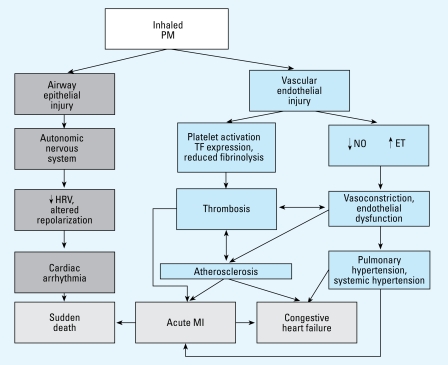
Mechanistic pathways for PM cardiovascular effects. Abbreviations: ET, endothelin; MI, myocardial infarction; NO, nitric oxide; TF, tissue factor. Modified from Frampton et al. 2009 (in press) with permission from Wolters Kluwer.

**Figure 2:**
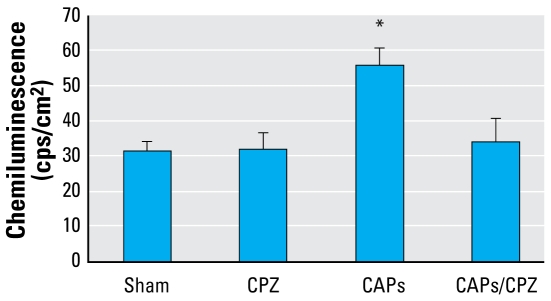
Capsazepine (CPZ) aerosolization prevents oxidative stress and damage in the heart of rats exposed to CAPs. Adult Sprague-Dawley rats received aerosols containing either 500 μM CPZ or saline for 20 min immediately prior to exposure to CAPs. Values represent the mean of eight independent determinations ± SEM. Reproduced from Ghelfi et al. (2008) with permission from Society of Toxicology. *p < 0.05.

The PM Centers convened a workshop to discuss potential mechanisms of PM-associated cardiovascular effects and to identify fruitful research approaches [Frampton et al. 2009 (in press; Utell et al. 2002] (Figure 1). During the first 6 years, center investigators have contributed to several review papers on cardiovascular responses to inhaled UFP and PM_2.5_ (Brook et al. 2004; Delfino et al. 2005; Godleski 2006; Mar et al. 2006; Pope and Dockery 2006). New statistical methodology was developed and applied to strengthen the interpretation of acute mortality studies (Coull et al. 2001; Janes et al. 2005; Schwartz and Coull 2003; Zanobetti et al. 2000, 2001; Zeka and Schwartz 2004). Epidemiologic studies that focused on specific cardiovascular outcomes, such as myocardial infarction (Peters et al. 2001, 2004; Zanobetti and Schwartz 2005) or cause-specific mortality (Franklin et al. 2007; Miller et al. 2007; Pope et al. 2002; Zeka et al. 2005) produced hypotheses for testing in laboratory animal research and human clinical studies. Toxicologists have contributed by identifying cellular and biomolecular mechanisms involved in the cardiovascular effects that result from acute and long-term exposures to ambient PM (Araujo et al. 2008; Corey et al. 2006; Lippmann et al. 2005a, 2006; Sun et al. 2005). Most recently, toxicologic studies (Ghelfi et al. 2008) have shown that increases in reactive oxygen species (ROS) in the heart associated with inhalation of concentrated ambient particles (CAPs) may be abrogated by blocking neural receptors in the lung (Figure 2).

Investigations in the PM Centers and elsewhere supported the hypothesis that inflammatory responses contribute to cardiovascular toxicity. Possible mechanisms were proposed. Pulmonary inflammation could release ROS, cytokines, and chemokines from the lung to the systemic circulation (Frampton et al. 2006b). Vascular inflammatory markers were associated with PM_2.5_ exposure in a subchronic mouse study (Sun et al. 2005). Gong et al. (2007), which demonstrated that both diesel extract and oxidized lipid components synergistically affect the expression profile of several gene modules related to vascular inflammatory processes. Evidence for an increase in C-reactive protein and a shift to a procoagulatory state of the blood was seen in coronary artery disease patients exposed to various size fractions of PM (Rückerl et al. 2006). Temporal and other parameters differed with the specific air pollution mixture in this study, which limited interpretation. Pope et al. (2004) concluded that fine particulate air pollution is a risk for cause-specific cardiovascular disease mortality via inflammation, accelerated atherosclerosis, and altered autonomic function. Zeka et al. (2006) reached similar conclusions. Their epidemiologic study supports the hypothesis that particles can induce cardiovascular disease through inflammatory pathways and suggests greater toxicity of traffic-related particles.

Autonomic function effects manifested as alterations in heart rate and heart rate variability (HRV) have been associated with PM_2.5_ exposure. Decreased HRV was associated with PM_2.5_ exposure in panel studies of elderly subjects (Adar et al. 2007; Henneberger et al. 2005; Schwartz et al. 2005a). No associations with altered heart rate or HRV were seen in Seattle during the winter woodburning season (Mar et al. 2005b; Sullivan et al. 2005). A population-based study that drew on an established cohort (the Normative Aging Study) confirmed the association between decreased HRV and PM_2.5_ seen in other studies; history of ischemic heart disease, hypertension, and diabetes modified the effects of PM_2.5_ (Park et al. 2005). Cardiac arrhythmias and vascular changes such as endothelial cell responses and alterations in blood pressure are other important clinical signs of cardiovascular toxicity that have been identified in both humans and animals exposed to PM (Frampton et al. 2006b; Gong et al. 2004; Nadziejko et al. 2002). Atherosclerosis is emerging as an important toxic end point of PM_2.5_ exposure.

**Figure 3:**
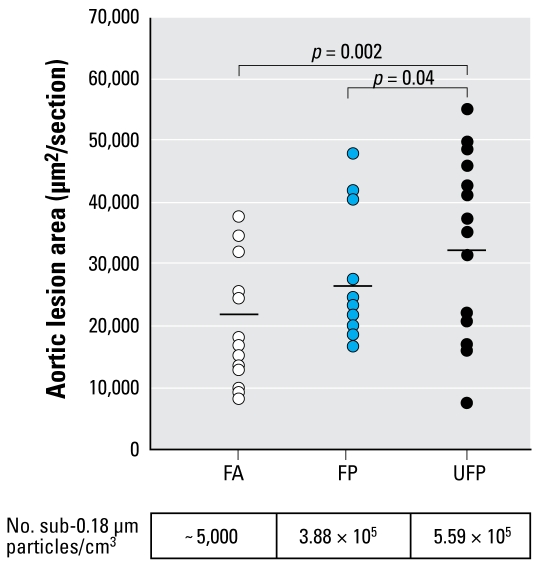
UFP is the most proatherogenic fraction. Atherosclerotic lesions were quantitatively analyzed in serial aortic root sections and stained with oil red O. Lesional area was scored as square micrometers per section and averaged ≥ 25 sections per animal. Group averages are indicated by straight horizontal bars. One mouse exposed to filtered air (FA) was an obvious outlier in its group and was removed from the atherosclerotic lesion analysis. However, its inclusion did not modify the overall significance. Mice exposed to FA are represented by white circles (*n* = 14), fine particles (FP) by blue circles (*n* = 16), and UFPs by black circles (*n* = 15). Reproduced from Araujo et al. (2008) with permission from Wolters Kluwer.

Atherosclerosis findings may be related to reports of myocardial infarction associated with PM_2.5_ in epidemiologic studies (Peters et al. 2004; Zanobetti and Schwartz 2005). The Peters study relates traffic exposures and myocardial infarction. Atherosclerotic lesions in a susceptible mouse model were enhanced by PM_2.5_ exposure in a number of reports (Araujo et al. 2008; Chen and Hwang 2005; Chen and Nadziejko 2005; Lippmann et al. 2005b; Sun et al. 2005). Araujo et al. (2008) compared the proatherogenic effects of ambient UFP with PM_2.5_ in apolipoprotein E–deficient mice. UFP-exposed mice exhibited significantly larger atherosclerotic lesions than mice exposed to PM_2.5_ or filtered air (Figure 3).

### Respiratory effects of PM exposure

PM Centers research has added to a wide body of literature investigating toxicologic mechanisms and effects of PM in the respiratory system. Overall, the issue of respiratory effects and PM exposure has been reviewed recently with reference to work produced by the PM Centers as well as others (Boothe and Shendell 2008; Salam et al. 2008). Salam focuses on asthma, whereas the Boothe and Shendell paper addresses some other end points in addition to respiratory effects. Results from clinical and panel studies in asthmatic and elderly subjects, as well as experimental studies in animals and *in vitro* cellular systems with relevance to respiratory tissues were reported. The discovery that UFP deposition is increased in asthmatic subjects during exercise has important implications for defining populations at greater risk of PM-related effects (Chalupa et al. 2004; Daigle et al. 2003). Adjuvant effects of ambient PM in promoting allergic airways responses occurred in a sensitized mouse model (Kleinman et al. 2005). Acute exposures to ambient PM in Seattle were associated with increased inflammation in asthmatic subjects, as measured by exhaled nitric oxide (Jansen et al. 2005; Koenig et al. 2005; Mar et al. 2005a). Respiratory effects in children were also a focus. Increased risk of infant hospitalization for bronchiolitis was significantly associated with subchronic and chronic exposures to PM in Los Angeles (Karr et al. 2007), where exposures in the month prior to hospitalization (subchronic) and mean lifetime exposure (chronic) referenced to the case diagnosis date were assessed on the basis of data derived from the California Air Resources Board. Epidemiologic studies that linked the PM Centers and the Children’s Health Study (CHS) contributed findings that identify infants and children as important populations of concern for respiratory effects of PM (Gauderman et al. 2004, 2005, 2007; Molitor et al. 2007; Trenga et al. 2006). These studies demonstrate that exposure to PM_2.5_ and other air pollutants were associated with reduced lung function growth in children and provided evidence for compromised lung function. The CHS/PM Center studies identified traffic as a risk factor (Gauderman et al. 2004, 2005, 2007; McConnell et al. 2006).

### Identification of new target tissues

**Figure 4:**
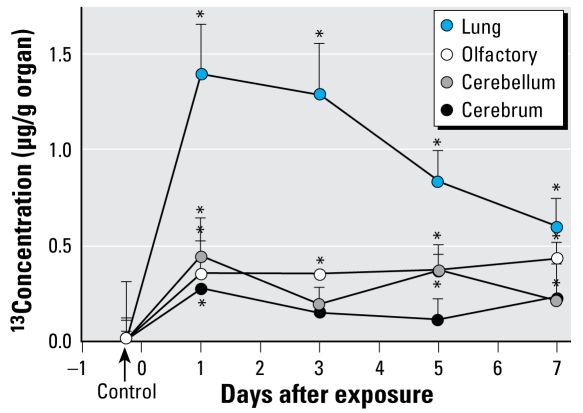
Time course of ^13^C tissue concentrations in lung, olfactory bulb, cerebrum, and cerebellum of rats after a 6-hr inhalation exposure to ultrafine (36 nm count median diameter) elemental ^13^C particles (*n* = 3 rats per time point). Adapted from Oberdörster et al. (2004) with permission from Taylor and Francis. *p < 0.05 (ANOVA).

UFP of carbon-13 were detected in the olfactory bulbs of rats after inhalation exposure (Oberdörster et al. 2004), suggesting that the central nervous system is a potentially important toxicologic target of PM_2.5_ (Figure 4). In support of this significant result, studies of mice chronically exposed to ambient PM_2.5_ documented loss of brain neurons (Veronesi et al. 2005) and changes in gene expression in the brain consistent with inflammatory effects (Gunnison and Chen 2005). In another study, proinflammatory cytokines were increased in brains of mice exposed to concentrated PM_2.5_ compared with those of control animals (Campbell et al. 2005).

### Chemical mechanisms of PM toxicity

To better identify the most toxic PM components and sources, the PM Centers have pursued experimental linkages between toxicologic properties and specific physical/chemical characteristics of particles including size, surface area, and PM components such as transition metals, endotoxin, and organics including reactive organic compounds. Multiple chemical and biological mechanisms by which PM can induce toxic effects in a variety of target cell types have been proposed (Frampton 2006; Yang et al. 2008). Oxidative stress, a common effect of toxicant exposure, is a change in the redox environment of the cell (Schafer and Buettner 2001) through changes in the ratios of concentrations of oxidized to reduced cellular antioxidants. Oxidative stress occurs by increasing intracellular ROS or by depleting glutathione (GSH). GSH is the predominant antioxidant in cells and plays important roles in protecting against oxidative and electrophile stress (Rahman and MacNee 2000). A number of PM Center studies during the first 6 years contributed to what is now a strong evidentiary basis for oxidative damage as a general toxicologic mechanism of PM injury (Delfino et al. 2005; Ghelfi et al. 2008; González-Flecha 2004; Gurgueira et al. 2002; Li et al. 2003a, 2003b; Rhoden et al. 2004, 2005; Tao et al. 2003; Xia et al. 2006). There is widespread agreement throughout the PM Centers that oxidative stress may be a mechanism of major importance for cardiorespiratory effects.

Studies of reactive chemical components of ambient PM samples reported that particles possess intrinsic chemical reactivity that may play an important role in toxicity (Cho et al. 2005; Venkatachari et al. 2005). Covalent modification of biological molecules by reactive electrophilic compounds, particularly organics, and ROS production are two key chemical mechanisms by which PM can disrupt intracellular biochemistry, ultimately altering gene expression and subcellular organelle function in target cells. Center investigators demonstrated covalent binding of a cellular enzyme by electrophilic agents, including organic compounds, present in ambient PM (Rodriguez et al. 2005; Samet et al. 1999) and reported that PM can directly inhibit the activity of enzymes involved in oxidative stress response in a cell-free assay (Hatzis et al. 2006). There is accumulating evidence that transition metals such as copper, vanadium, chromium, nickel, cobalt, and iron, as well as aromatic and polar organic substances, play a role in ROS production. An important role of metals may be alteration of signal transduction pathways involving oxidative stress (Samet et al. 2003). Assays that can screen for both oxidative and covalent binding properties of PM are of interest for comparing the toxicologic potential of PM from different sources, locations of interest, season, and other parameters of interest (Borm et al. 2007).

### Life shortening associated with exposure to PM

In analyses at the Harvard Center in which daily deaths in 10 European cities were investigated by examining all-cause, respiratory, and cardiovascular deaths for all ages and stratifying by age groups, it was found that the effect of air pollution is not limited to advancing mortality by a few weeks, but that effects persist for over a month after exposure. The short-term mortality effect size estimate for PM_10_ doubles when longer-term effects for all mortality and cardiovascular mortality are considered and becomes five times higher for respiratory mortality (Zanobetti et al. 2003). Reduction of ambient air pollution levels was associated with reduced total, cardiovascular, and lung cancer mortality in the Harvard Six Cities Cohort (Laden et al. 2006). Long-term exposure was associated with excess lung cancer in cohort studies of Pope et al. (2002), Laden et al. (2006), and Pope and Dockery (2006).

### Susceptibility factors and populations of concern for PM-induced health effects

When the PM Centers research was initiated, epidemiologic studies had indicated that the elderly and people with cardiovascular or chronic lung disease were at greater risk for morbidity and mortality associated with acute PM exposure. The PM Centers explored the basis for this susceptibility and also produced research findings that expand the spectrum of populations of concern. Support for the epidemiologic observations that elderly and chronic obstructive pulmonary disease patients have higher rates of hospitalization and mortality associated with acute PM exposure has come from human clinical studies showing that elderly people experience greater effects of PM on HRV and blood parameters (Park et al. 2005; Pope and Dockery 2006; Schwartz et al. 2005a, 2005b). Further support for the elderly as a population of concern comes from studies of geriatric laboratory animals (Elder et al. 2004a, 2004b).

A study of PM-related daily mortality found greater effects in diabetic subjects (Zeka et al. 2006). The increase in mortality in diabetics may be related to increased susceptibility to the cardiovascular effects of PM exposure, as indicated by greater rate of hospitalization for heart disease (Zanobetti and Schwartz 2002), sensitivity to changes in HRV (Park et al. 2005), and altered vasomotor function (O’Neill et al. 2005) in diabetic subjects. It is possible that these patients may be more susceptible to inflammatory effects of PM, which in turn affect vascular tissues (O’Neill et al. 2007). In contrast, recent results from the Women’s Health Initiative suggest that diabetics in this cohort were not at increased risk (Miller et al. 2007). More work on this subject is needed, and controlled human exposures in diabetic studies have been initiated by the PM Centers (Frampton et al. 2006a). Schwartz et al. (2005b) reported an association between presence or absence of the allele for glutathione-*S*-transferase M1 and the high frequency component of HRV. Genetic susceptibility is an area in which the PM Centers are currently increasing research focus.

## Advances in Critical Interdisciplinary Research Areas

Interdisciplinary research has been a hallmark of the PM Centers since their inception. Two subject areas that were exemplary in terms of bringing together multiple investigative perspectives were investigations of UFP and mobile sources.

### Ultrafine particles: unique in composition and toxicity

Center-based research allowed a major effort to characterize size distributions, chemical speciation, and the effect of atmospheric processes of UFP to be integrated with toxicologic research (Donaldson and Stone 2003). UFP in urban airsheds are largely derived from fresh combustion sources, although secondary formation of UFP from atmospheric photochemical processes is also an important source (Sioutas et al. 2005). UFP freshly generated by combustion are short-lived and subsequently grow to form aggregates. UFP dominate particle number concentration in ambient PM samples while contributing little to PM mass concentrations. In part because of a complex fractal structure (Friedlander and Xiong 2000), UFP possess much greater surface area per unit mass than larger ambient particles. The large surface area, in turn, allows greater per-mass concentrations of adsorbed or condensed toxic air pollutants (oxidant gases, organic compounds, transition metals) to collect on UFP (Sioutas et al. 2005). Studies on ambient and model particles have concluded that the large specific surface area of UFP may be a key component in their toxicology (Oberdörster 2001).

The PM Centers produced an integrated body of exposure and toxicologic studies on ambient and model UFP as well as studies of controlled human exposures. Dosimetry work showed that UFP will have significant accumulation in the lung (Kreyling et al. 2006). In addition, UFP of varying composition can cross cellular membranes by diffusion (Geiser et al. 2005) and gain access to vulnerable targets within cells. The potential for translocation from the site of lung deposition into systemic circulation, although rates have been low with test particles (Kreyling et al. 2002), could have major mechanistic implications (Elder and Oberdörster 2006). Electron microscopy indicated subcellular penetration and mitochondrial damage by UFP in *in vivo* studies and, to a lesser extent, by fine particles (Li et al. 2003b). Disruption of mitochondrial functions may play an important role in PM-mediated health effects (Xia et al. 2007).

In a study of size-segregated concentrated ambient PM samples, the ability of PM to catalyze ROS generation, an initial step in the induction of oxidative stress, was greatest in the UFP fraction (Cho et al. 2005). Li et al. (2003a) summarized contrasting features of coarse, fine, and ultrafine particles from Southern California, including relevant chemical and biological parameters. The toxicologic findings correlated with PM OC and polycyclic aromatic hydrocarbon (PAH) composition, suggesting a role of organic agents in generating redox activity (Table 1).

The PM Centers conducted controlled human exposure studies with UFP. Results from these studies were limited, because of small group sizes and because these exposures are necessarily brief and conducted at low concentrations compared with the background PM exposures that may be experienced by urban study subjects. In the first set of studies, short-term exposures were conducted with 10–50 μg/m^3^ carbon UFP generated in the laboratory. Alterations in blood cell adhesion molecules and in a marker of vascular perfusion suggest that UFP exposure may produce subtle changes in pulmonary vasoconstriction (Frampton 2007; Pietropaoli et al. 2004). A small but statistically significant reduction in arterial oxygen saturation and some evidence for reduced HRV were found, although the small study size limited interpretation (Gong et al. 2008). An expanded focus on UFP in epidemiologic studies is needed but has been limited to date by the challenges of assessing exposure to UFP.

### Traffic: mobile sources are highly relevant to the public health impacts of PM

The center-based research context was particularly useful in advancing the science on mobile sources of PM, the focus of an extensive international research effort. Numerous investigations of the physical and chemical attributes of PM collected alongside freeways and in roadway tunnels were performed. The results have yielded data on size distribution, number and mass concentrations, chemical speciation, emissions factors, volatility, penetration indoors, and the impact of atmospheric processes on roadway PM (Biswas et al. 2007; Fine et al. 2004; Geller et al. 2006; Kuhn et al. 2005b, 2005c; Phuleria et al. 2007; Sardar et al. 2005; Zhu et al. 2005). Detailed spatial profiles of UFP concentration at varying distances from freeways were generated (Zhu et al. 2002a, 2002b). Concentrations of UFP drop exponentially with distance from the center of the freeway, reaching upwind levels at approximately 300 meters. The size distribution of UFP also changed markedly with distance reflective of coagulation and other atmospheric particle processes. Winter particle number concentrations are greater than summer, indicating formation of UFP from vapor condensation. Exposure to motor vehicle exhaust emissions during commuting may constitute a substantial fraction of daily personal PM exposure, especially to UFP (Sioutas et al. 2005; Zhu et al. 2007).

Toxicologic studies of traffic-derived aerosols studied by PM Centers included *in vitro* findings that implicate PM collected in freeway microenvironments in the production of reactive chemical species, stimulation of proinflammatory effects, and altered gene expression in cellular test systems. UFP fraction, carbonaceous content, and an organic tracer for vehicles were linked with toxicologic activity of PM in a variety of assays (Cho et al. 2005; Li et al. 2003a, 2003b). Several studies of laboratory animals exposed to PM on or near busy roadways have identified cardiovascular and allergic airways effects (Elder et al. 2004b, 2007; Kleinman et al. 2005). Evidence that traffic-derived air pollution affects humans has expanded significantly during the first 6 years of PM Centers funding, implicating mobile source in respiratory effects in children (Gauderman et al. 2004, 2005, 2007; McConnell et al. 2006), cardiovascular effects (Riediker et al. 2004) including myocardial infarction (Peters et al. 2004; Tonne et al. 2007), and low birth weight (Wilhelm and Ritz 2003). Toxicologic studies are needed to follow up the epidemiologic findings of effects on the fetus. In a reanalysis of data from the Harvard Six Cities study of daily mortality and PM, source apportionment approaches identified the mobile source factor as most strongly associated with increased daily mortality (Laden et al. 2000).

## Policy Implications of PM Centers Research

Research findings from the PM Centers have had a significant influence on science policy, most directly in terms of the science that underlies the National Ambient Air Quality Standards (NAAQS) for PM. The findings of morbidity and mortality that form the scientific basis for the short-term and annual PM NAAQS were strengthened through epidemiologic and statistical research. Mechanistic investigations and studies of preclinical markers established biological plausibility for observed relationships between ambient air PM and observed acute mortality. In personal exposure studies, validation of the use of central site ambient concentrations provided crucial support to the interpretation of epidemiologic results.

The PM NAAQS are based on mass concentration. The state of the science suggests that no single parameter, whether mass, size fraction, surface area, or a particular chemical component, is responsible for all the diverse mechanisms and toxicologic end points that have been associated with PM, and a more sophisticated approach to standards will be needed. Based on findings from the PM Centers and others, the potential efficacy of number and component based standards should be assessed. As more data become available to link specific PM emissions sources, chemical composition, and physical characteristics with quantitative measures of toxicity, the question of source-specific control strategies to maximize public health protection also needs to be considered.

The increasing level of evidence that UFP are toxic but may not be controlled well by existing regulatory approaches raises other policy issues including mitigation of the risk of health effects associated with housing, schools, parks, and other heavily populated public facilities located near heavily traveled roadways, busy seaports, and other combustion sources that are the major urban sources of exposure to UFP. There are potential environmental justice concerns associated with transportation-derived combustion, as it is often areas of lower socioeconomic status that are most affected by proximity to these sources.

## Looking Forward: Research Priorities and Current Directions

As the PM Centers program moved forward into the second phase, the original guiding research priorities were reevaluated, and new priorities have emerged. Several areas of investigation identified during the development of the 1997 PM NAAQS are still of critical relevance today, but the scientific questions being asked have been refined. Some research topics being pursued in the current round of PM Centers are described below.

### Particle source characterization and PM components as factors in PM toxicity

The PM Centers current research agenda includes detailed studies of the physical and chemical attributes of ambient PM associated with specific sources. The current science indicates that multiple mechanisms of injury, in backgrounds modified by host susceptibility factors, can be activated by a variety of PM components and characteristics. To address the complexity associated with assessing the health effects associated with specific PM components, the current PM Centers research agenda compares toxicologic properties of PM by source type in addition to compositional attributes. Mobile sources continue to be a priority focus, and there is a need to better understand the fate of fossil fuel combustion emissions from a variety of mobile and stationary sources, including airports, seaports, and other sources as well as roadways. Building upon the productive body of work on mobile source PM in the first 6 years of PM Center work, the current PM Centers include human panel and clinical studies and toxicologic studies in laboratory animals and *in vitro* systems that test hypotheses about the effects of mobile source PM exposures. Source apportionment efforts are ongoing as well, to build on previous work that found mobile sources are dominant contributors to urban UFP loads. *In vitro* studies will pay particular attention to UFP, organic compounds, and transition metals. UFP formed from nucleation of ambient air vapors are a new focus, as they may be especially toxic.

### Dosimetry and toxicokinetics

Research at the PM Centers is addressing particle deposition, uptake, distribution, and fate, including the effects of developmental stage on disposition of PM. Cell culture systems with gene expression and proteomics methods are being used for studies of metabolic and genetic responses that will be useful for toxicokinetics. Studies of the dosimetry and toxicokinetics associated with UFP are especially important, given previous PM Centers findings that these particles distribute into systemic circulation and secondary target organs such as the CNS, and can enter cells and subcellular organelles.

### Mechanisms

All the current PM Centers have a strong focus on continuing to develop understanding of the toxic mechanisms that underlie clinically and epidemiologically defined adverse health effects of PM. Mechanisms being pursued include reactive chemical species that cause cellular oxidative stress responses. In the first 6 years, studies of oxidative damage associated with PM were performed using diverse chemical species, cell culture experiments, and laboratory animal studies. Evolving from that work, the current PM Centers studies are looking at markers of oxidative stress processes in humans and a range of clinical and preclinical biomarkers. The list of gene products that can be used as indicators of PM exposure or toxicity in various cell types has expanded. Mechanistic hypotheses are being tested in panel and other epidemiologic studies.

### Susceptibility

Susceptibility is a major theme, drawing on the work from the earlier center and noncenter investigators showing that individuals with pulmonary and cardiac health conditions, elderly, children, diabetics, and others may be more susceptible to the adverse effects of PM exposure than the general population. The PM Centers are looking at early life exposures to PM in animal models, performing panel studies of elderly subjects or subjects with compromised health status, using a large established cohort to identify how risk factors for PM-related health outcomes may be modified by individual factors such as medication use, diet, and genotype. Compromised animal models are a key theme of current research into susceptibility. PM exposure studies on ApoE^−/−^ mice (an atherosclerosis-prone model), hypertensive rats, and diabetic rats are all planned or underway.

## Conclusions

In 1998, a committee of the NRC published the first of a four-volume report titled “Research Priorities for Airborne Particulate Matter” that identified the 10 highest-priority targets for PM research (NRC 1998). Within the research portfolio of the PM Centers, the priority areas have been addressed. A subsequent NRC report (2001) emphasized that these research priorities require multidisciplinary approaches. Recognizing that progress in understanding the health effects consequent to air pollution exposure requires talents from highly divergent fields, we believe that the PM Centers effectively promote interdisciplinary cross-fertilization. The next 5 years of this program will bring the experience and results of the first centers to fruition in new, focused studies that we hope will be instrumental in addressing the difficult scientific and public health policy problems that arise from ubiquitous particulate air pollution.

## Correction

In the title of the manuscript originally published online, the date range in the title was incorrect. It has been corrected here.

## Figures and Tables

**Table 1:** Contrasting features of coarse, fine, and ultrafine particles.

Parameters	Coarse PM_10_	Fine PM_10_	Ultrafine PM_10_
Size (μm)	2.5–10	2.5–0.15	< 0.15
OC content	+	++	+++
EC content	+	++	+++
Metals (% of total elements)	+++	++	+
PAH content	+	+	+++
Redox activity (DTT assay)	+	++	+++
HO-1 induction	+	++	+++
GSH depletion	+	+++	+++
Mitochondrial damage	None	Some	Extensive

Data from Li et al. (2003a).
